# The influence of real-time blood glucose levels on left ventricular myocardial strain and strain rate in pediatric patients with type 1 diabetes mellitus - a speckle tracking echocardiography study

**DOI:** 10.1186/s12872-015-0171-5

**Published:** 2015-12-21

**Authors:** Kai O. Hensel, Franziska Grimmer, Andreas C. Jenke, Stefan Wirth, Andreas Heusch

**Affiliations:** Department of Pediatrics, HELIOS Medical Center Wuppertal, Center for Clinical & Translational Research (CCTR), Faculty of Health, Center for Biomedical Education & Research (ZBAF), Witten/Herdecke University, Faculty of Health, Heusnerstr. 40, D-42283 Wuppertal, Germany

**Keywords:** Speckle tracking echocardiography, Two-dimensional strain, Strain rate, Transient metabolic changes, Blood sugar, Diabetic cardiomyopathy, Systolic function, Subclinical LV impairment, Normal ejection fraction

## Abstract

**Background:**

Echocardiographic myocardial performance parameters such as strain and strain rate are increasingly used to assess systolic and diastolic function in patients with diabetes mellitus and several other clinical and scientific scenarios. While long-term metabolic marks such as HbA_1C_ are inherently assessed in diabetic patients, the actual blood glucose level at the very moment of the echocardiographic study has not yet been taken into account for the assessment of cardiac mechanics. The aim of this study was to investigate the influence of real-time blood glucose levels on left ventricular (LV) myocardial strain and strain rate in pediatric patients with type 1 diabetes mellitus (T1DM).

**Methods:**

We performed speckle tracking echocardiography on 39 normotensive pediatric patients with uncomplicated type 1 diabetes mellitus (mean age 11.5 ± 3.5 years, 40 % female) and 44 sex- and age-matched healthy controls (mean age 11.4 ± 2.9 years, 45 % female). T1DM patients were sub-categorized according to their blood sugar levels (with a cutoff of 150 mg/dL) at the moment of the echocardiographic exam. Investigators were blinded to the participants’ study group status.

**Results:**

Interestingly, diabetic patients with higher blood sugar levels demonstrated significantly increased LV circumferential strain (*p* = 0.003) and strain rate (*p* = 0.005) as well as global longitudinal strain rate (*p* = 0.002) in comparison to T1DM patients with lower blood sugar levels or healthy controls.

**Conclusions:**

For the investigation of myocardial performance with sensitive methods such as speckle tracking echocardiography in diabetic study populations real-time blood sugar levels should be taken into account. Further studies are needed to verify these findings in large-scale patient cohorts and serial intra-individual measurements in different metabolic states.

## Background

Quantitative echocardiographic assessment of regional and global myocardial deformation –strain and strain rate - is increasingly used for the evaluation of systolic and diastolic function in diabetic cardiomyopathy and in a variety of other scientific and clinical scenarios [1–3]. In health and disease, left ventricular (LV) cardiac mechanics are complex and heterogeneously variable in nature. Hence, sensitive LV performance measurements in diabetes associated cardiac disease are susceptible to alterations due to advanced age, disease duration (Kai O. Hensel, Franziska Grimmer, Markus Roskopf, Andreas C. Jenke, Stefan Wirth, and Andreas Heusch, “Subclinical Alterations of Cardiac Mechanics Present Early in the Course of Pediatric Type 1 Diabetes Mellitus: A Prospective Blinded Speckle Tracking Stress Echocardiography Study,” Journal of Diabetes Research, Article ID 2583747, in press), diabetic complications [4], overweight [5], inotropically active drugs [6], etc.. However, there is a total lack of studies on the effect of short-term alterations in serum glucose levels in pediatric patients with diabetes mellitus. Children and adolescents with uncomplicated diabetes may serve as an ideal model to study the effect of blood glucose levels on myocardial function in the absence of potentially confounding factors such as ischemia or renal disease. Variations in serum glucose are known to significantly affect myocardial contractility through several molecular mechanisms [7, 8]. For logistical reasons blood sample collection and echocardiographic examinations oftentimes are performed separately in clinical studies, sometimes with days or even weeks in between. Alterations in the concentration of intravenous lipids have been shown to have an immediate effect on myocardial contractility [9]. However, serum glucose levels at the very moment of the echocardiographic assessment have not yet been taken into account when analyzing myocardial mechanics in diabetic patients. The aim of this study was to investigate the influence of real-time blood glucose on the echocardiographic LV performance parameters strain and strain rate in pediatric patients with uncomplicated type 1 diabetes mellitus.

## Methods

In order to study the effect of real-time blood sugar levels on LV myocardial deformation we utilized speckle tracking echocardiography to measure LV myocardial strain and strain rate as previously described [10]. We included 83 children and adolescents; 39 consecutive asymptomatic, normotensive children and adolescents (40 % female) with uncomplicated type 1 diabetes mellitus (T1DM) and 44 age- and sex-matched healthy controls (45 % female). Exclusion criteria were any signs of diabetes associated end-organ damage (e.g. proteinuria) as well as other medical conditions that may affect the cardiovascular system. None of the included patients or volunteers were on any medication with influence to the cardiovascular system (other than insulin for the study group). Echocardiographic examinations were performed in a left lateral decubitus position according to a standardized protocol using the commercially available ultrasound device iE33 by Phillips Ultrasound Inc., USA, with a S5-1 Sector Array transducer (Sector 1-5 MHz). A complete standard 2D study including a spectral and color flow Doppler examination was carried out according to international echocardiography guidelines [11]. Three consecutive cardiac cycles were recorded as digital loop for subsequent quantitative analyses on an off-line work station. XCelera Version 3.1.1.422 by Phillips Ultrasound Inc., USA was used for speckle-tracking derived strain and strain rate measurements. Circumferential strain and strain rate were assessed in the parasternal short axis view at the level of the mitral valve and the papillary muscles. Global longitudinal strain and strain rate were derived by measuring cardiac deformation in apical 2-, 3-, and 4-chamber views. A priori the diabetes group was sub-categorized according to their blood sugar level at the moment of the echocardiographic examination in groups with blood glucose levels lower or higher than 150 mg/dL, respectively. For the quantitative deformation post processing of echocardiographic images the investigators were blinded to the group status of the participants.

The study was approved by the Witten/Herdecke University Ethics and Clinical Trials Committee and assigned the trial number 113/2013. Each participant as well as their legal guardian signed a written informed consent. For ethical reasons it was not feasible to obtain blood chemistry parameters including serum glucose levels from those healthy children who voluntarily served as a control group.

Data is presented as mean (± standard deviation) in tables and in box-whisker-plots with median and interquartile ranges. One way analysis of variance (ANOVA) and post hoc (Turkey) test as well as Mann-Whitney *U* test and post hoc bonferroni correction were used to find statistically significant differences among the groups. According to post hoc bonferroni correction the statistical significance level was set to 0.00625 for strain and strain rate analyses. SPSS Version 22.0 and Microsoft Excel for Mac 2011 Version 14.4.4 were utilized for statistical analyses and data presentation.

## Results

Baseline clinical characteristics and conventional echocardiographic parameters almost invariably did not differ significantly between the analyzed groups (Tables 1 and 2). Mean age was 11.5 ± 3.5 years in the high blood sugar diabetes group, 11.5 ± 3.3 years in the low blood sugar diabetes group and 11.4 ± 2.9 years in the controls. Mean disease duration (and HbA_1c_) were 4.5 ± 3.0 years (8.3 ± 1.2 mg/dL) in T1DM patients with high blood sugar and 4.5 ± 3.3 years (8.1 ± 1.2 mg/dL) in T1DM patients with low blood sugar at the moment of the echocardiographic examination. Exceptions are a slightly higher but normal heart rate in T1DM patients with blood sugar levels > 150 mg/dL (87.1 ± 11.4 versus 81.3 ± 10.8 bpm) and a greater end-systolic interventricular septal diameter (1.17 ± 0.20 vs. 1.09 ± 0.21 cm) and LV mass (115.2 ± 37.6 vs. 100.3 ± 41 g) in healthy controls. Even though statistically significant these differences were marginal and all values were within normal limits as evaluated by z-scores [12].

**Table 1:** Baseline clinical characteristics and hemodynamics of the study population

	Type 1 diabetes mellitus		Control	*p*-value
	Blood glucose level < 150 mg/dl	Blood glucose level > 150 mg/dl	(*n* = 44)	
	(*n* = 17)	(*n* = 22)		
Age (years)	11.5 ± 3.3	11.5 ± 3.0	11.4 ± 2.9	n.s.
Height (cm)	152.2 ± 19.5	152.8 ± 17.5	154.1 ± 16.8	n.s.
Weight (kg)	44.6 ± 16.2	47 ± 16.2	48.0 ± 16.3	n.s.
Body surface (m^2^)	1.37 ± 0.3	1.41 ± 0.3	1.4 ± 0.3	n.s.
Body mass index (kg/m^2^)	18.5 ± 2.3	19.5 ± 3.2	19.6 ± 3.5	n.s.
Exercise routine (1–3)	1.8 ± 0.8	1.7 ± 0.8	2.0 ± 0.7	n.s.
Diabetes duration (years)	3.2 ± 3.8	4.5 ± 3.3	-	n.s.
HbA_1c_ (%)	8.3 ± 1.2	8.1 ± 1.2	-	n.s.
Heart rate (beats/minute)	81.3 ± 10.8	87.1 ± 11.4	76.2 ± 9.4	0.001
BP systolic (mmHg)	106.3 ± 7.5	104.7 ± 10.9	105.8 ± 9.2	n.s.
BP diastolic (mmHg)	58.2 ± 7.8	58.5 ± 8.2	59.4 ± 9.2	n.s.

Exercise routine level: 1 = in school; 2 = < 3 times/week; 3 = ≥ 3 times/week, *p*-values calculated with one way ANOVA, post hoc and Mann-Whitney *U* test, level of significance = 0.05

**Table 2:** Conventional echocardiographic parameters derived from two-dimensional and Doppler imaging

	Type 1 diabetes mellitus		Control	*p*-value
	Blood glucose level < 150 mg/dl	Blood glucose level > 150 mg/dl		
	(*n* = 17)	(*n* = 22)	(*n* = 44)	
Aortic root (AoR) diameter (cm)	2.28 ± 0.36	2.35 ± 0.33	2.41 ± 0.35	n.s.
Left atrial (LA) diameter (cm)	2.48 ± 0.37	2.58 ± 0.36	2.71 ± 0.45	n.s.
LA/AoR	1.10 ± 0.17	1.11 ± 0.16	1.13 ± 0.16	n.s.
Fractional shortening (%)	33.03 ± 4.48	33.70 ± 3.93	34.78 ± 3.94	n.s.
End-systolic interventricular septal diameter (cm)	0.99 ± 0.21	1.09 ± 0.21	1.17 ± 0.20	0.018
End-diastolic interventricular septal diameter (cm)	0.82 ± 0.16	0.85 ± 0.18	0.89 ± 0.16	n.s.
LV end-systolic diameter (cm)	2.72 ± 0.49	2.66 ± 0.39	2.76 ± 0.41	n.s.
LV end-diastolic diameter (cm)	4.05 ± 0.63	4.01 ± 0.49	4.27 ± 0.46	n.s.
End-systolic LV posterior wall diameter (cm)	1.25 ± 0.18	1.21 ± 0.23	1.27 ± 0.21	n.s.
End-diastolic LV posterior wall diameter (cm)	0.80 ± 0.16	0.78 ± 0.17	0.81 ± 0.15	n.s.
Left ventricular mass (g)	102.49 ± 42.90	100.28 ± 41.01	115.18 ± 37.56	0.02
Relative wall thickness	0.20 ± 0.05	0.19 ± 0.03	0.19 ± 0.03	n.s.
LV end-diastolic volume (ml)	65.84 ± 28.13	72.72 ± 22.11	79.63 ± 27.97	n.s.
LV end-systolic volume (ml)	25.96 ± 9.17	28.98 ± 10.12	31.66 ± 11.78	n.s.
Ejection fraction (%)	61.96 ± 4.20	61.14 ± 5.05	60.16 ± 4.67	n.s.
Stroke volume (ml)	44.0 ± 15.7	45.3 ± 14.1	49.3 ± 18.1	n.s.
Cardiac output (l/min)	3.5 ± 1.2	3.9 ± 1.0	3.7 ± 1.3	n.s.
Mitral inflow: E-Wave (cm/s)	98.77 ± 15.37	93.63 ± 11.19	96.86 ± 14.26	n.s.
Mitral inflow: A-wave (cm/s)	61.30 ± 14.76	60.62 ± 10.53	57.36 ± 10.41	n.s.
E-Wave / A-Wave	1.67 ± 0.32	1.57 ± 0.23	1.72 ± 0.26	n.s.
Mitral deceleration time (s)	0.17 ± 0.04	0.17 ± 0.04	0.18 ± 0.04	n.s.
Isovolumetric relaxation time (s)	0.05 ± 0.01	0.05 ± 0.01	0.05 ± 0.01	n.s.
S’ (cm/s)	7.74 ± 0.88	8.05 ± 1.05	8.17 ± 1.19	n.s.
E’ (cm/s)	12.81 ± 1.94	12.41 ± 1.73	13.03 ± 1.87	n.s.
A’ (cm/s)	5.24 ± 0.92	5.56 ± 1.41	5.51 ± 1.11	n.s.
E’/A’ (cm/s)	2.49 ± 0.47	2.42 ± 0.92	2.48 ± 0.72	n.s.
E/E’ (cm/s)	7.82 ± 1.28	7.65 ± 1.17	7.56 ± 1.42	n.s.

*p*-values are calculated with one way ANOVA and post hoc test, level of significance = 0.05

**Fig. 1:**
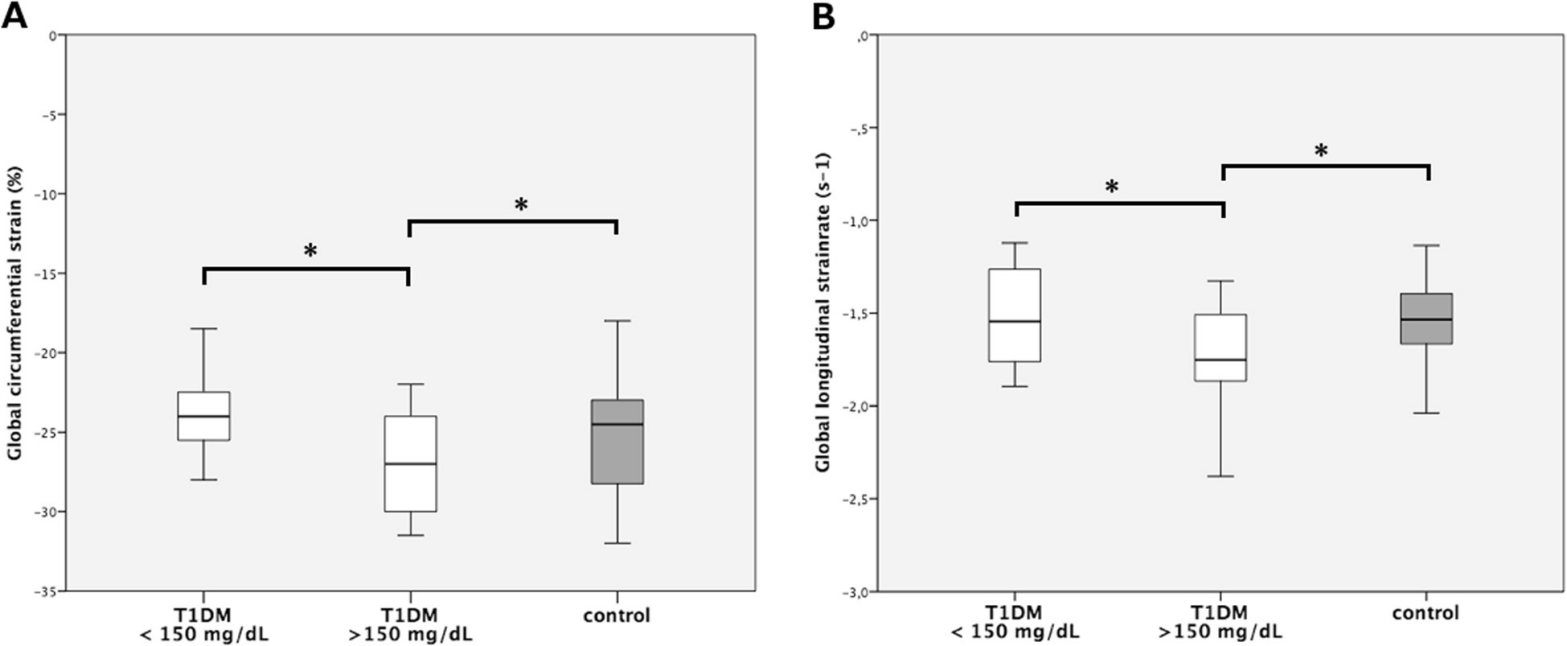
Myocardial deformation in type 1 diabetes mellitus patients and healthy controls in relation to serum glucose levels at the moment of the echocardiographic examination. **a**: LV peak systolic global circumferential *strain* in healthy controls and T1DM patients. **b**: LV peak systolic global longitudinal *strain rate* in healthy controls and T1DM patients. * = *p* < 0.00625; *p*-values were calculated with one way ANOVA and post hoc tests

**Fig. 2:**
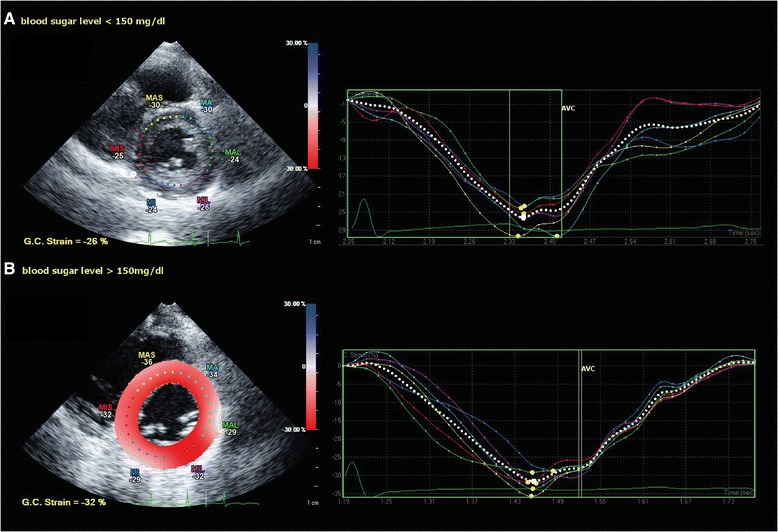
Speckle tracking echocardiography in the parasternal short axis view at level of the papillary muscles. **a** peak systolic global LV circumferential strain in a pediatric patient with type 1 diabetes mellitus and real time serum glucose level < 150 mg/dL. **b** peak systolic global LV circumferential strain in a pediatric patient with type 1 diabetes mellitus and real time serum glucose level > 150 mg/dL. *Note the increased (more negative) peak LV systolic global circumferential strain in the diabetic patient with the higher blood sugar level (* ***b*** *)*

The effect of real-time serum glucose levels on myocardial deformation parameters is demonstrated in Fig. 1. T1DM Patients with blood sugar levels higher than 150 mg/dL at the time of the echocardiographic examination had significantly increased (more negative) peak LV circumferential *strain* (*p* = 0.003), circumferential *strain rate* (*p* = 0.005) and longitudinal *strain rate* (*p* = 0.002) values than patients with lower real-time blood sugar levels or healthy controls (Table 3). The difference in global longitudinal *strain* showed the same tendency with increased values in T1DM patients with higher real-time blood sugar levels when compared to T1DM patients with lower blood sugar levels, however without reaching statistical significance. A speckle-tracking echocardiography example of increased global LV peak circumferential strain in a patient with elevated serum glucose is shown in Fig. 2. The results were reproducible and inter-observer variability was below 6 %.

**Table 3:** Speckle tracking echocardiography derived peak LV circumferential and longitudinal strain and strain rate of T1DM patients and healthy controls

	Type 1 diabetes mellitus		Control	*p*-value
	Blood glucose level <150 mg/dl	Blood glucose level >150 mg/dl		
	(*n* = 17)	(*n* = 22)	(*n* = 44)	
Circumferential strain	-24.24 ± 3.11	-28.81 ± 4.84	-25.95 ± 3.92	0.003
Circumferential strain rate	-1.96 ± 0.26	-2.12 ± 0.40	-1.86 ± 0.25	0.005
Global longitudinal strain	-19.55 ± 2.27	-20.57 ± 2.41	-20.72 ± 2.49	n.s.
Global longitudinal strain rate	-1.51 ± 0.26	-1.85 ± 0.51	-1.55 ± 0.21	0.002

*p*-values are calculated with one way ANOVA and post hoc test, level of significance = 0.00625

## Discussion

Myocardial performance is influenced by a variety of non-metabolic and metabolic factors [13]. When studying myocardial function in clinical and scientific scenarios, usually only long-term influences are taken into account [14]. Currently, there are no studies on the effect of short-term blood glucose alterations on cardiac mechanics in pediatric patients with diabetes mellitus. However, acute metabolic changes have been shown to substantially alter cardiac function. Holland et al. demonstrated, that the infusion of an intravenous fat emulsion to acutely raise plasma triglycerides alters ventricular-vascular interaction by increasing left ventricular contractility without affecting arterial load [9]. Recently, longitudinal strain was shown to be influenced by fasting plasma glucose levels in adult patients with type 2 diabetes mellitus [15]. To our knowledge the present study is the first clinical study in humans to assess the influence of real-time blood sugar levels on myocardial performance parameters strain and strain rate in a diabetic study population.

Interestingly, we found strain and strain rate to be increased (more negative) in diabetic children with elevated real-time blood sugar levels when compared to T1DM patients with lower blood sugar levels and healthy controls (Table 3, Fig. 1). This is in line with an *in vitro* finding of the acute influence of fructose on cardiomyocyte excitation-contraction coupling [16] – a molecular mechanism that might explain the results of the present study. The fact that circumferential strain, circumferential strain rate and longitudinal strain rate but not longitudinal strain were significantly higher in T1DM patients with increased blood sugar levels can well be explained by the strong heterogeneity of myocardial fiber organization in the LV and the natural consequence of regional variations in myocardial deformation [17, 18]. Hence, transient metabolic laboratory biochemistry parameters such as serum glucose levels may affect LV myocardial performance. Consequently, blood glucose and lipid studies should be obtained close to the time of the echocardiographic examination when assessing cardiac mechanics with sensitive tools such as speckle-tracking.

Baseline characteristics of the study groups did not differ significantly between the analyzed groups besides for a slightly higher heart rate in T1DM patients with blood sugar levels higher than 150 mg/dL (87.1 ± 11.4 versus 81.3 ± 10.8 bpm; Table 1). Even though statistically significant, this difference in heart rate is unlikely to be a biologically relevant cause for the above described alterations in myocardial strain and strain rate. Boettler and colleagues have found differences in heart rate mainly to affect strain rate in diastole other than in systole [19]. Furthermore, systolic strain has been shown to decrease with increasing heart rate [20]. This means, that if heart rate had a significant influence on strain and strain rate in our study population, it would actually lead to depressed strain values in T1DM patients with high blood sugar levels. Hence, the finding of increased strain in combination with a slightly higher heart rate in T1DM patients with high blood sugar levels even strengthens the significance of real-time blood glucose for myocardial performance parameters as demonstrated in this study. Nevertheless, it has to be stated that the here reported changes represent subtle, subclinical alterations only. As the study participants do not suffer from overt heart disease but exhibit uncomplicated T1DM with a relatively short disease duration (mean 4 ± 3.5 years) only, it is not surprising that the demonstrated differences in strain and strain rate are not more pronounced. Outcome-relation and clinical implications yet have to be determined by further studies. Hypothetically, hyperglycemia-induced increased energy turnover may lead to hyperdynamic cardiac mechanics in diabetes associated cardiomyopathy which on the long run might contribute to the development of diabetic cardiomyopathy. Experimental animal studies utilizing speckle tracking echocardiography in hyperglycemic ketoacidosis and hypoglycemic states should be performed to further illuminate the spatiotemporal course of diabetes associated cardiac disease.

Study limitations are the fact that this is an observational study in a limited number of patients. In order to correlate the findings of altered myocardial strain and strain rate with a clinical outcome, long-term follow-up studies in large-scale cohorts are necessary. These studies should include adult individuals and serial intra-individual echocardiographic assessments in states of different blood glucose levels both in diabetic and healthy control patients, which was not feasible for this study due to ethical restrictions. These further studies are needed to ultimately establish the value of real time glucose level measurements for the evaluation of the myocardial contractile state using sensible methods such as speckle-tracking echocardiography.

## Conclusion

LV myocardial strain and strain rate in pediatric patients with T1DM may be influenced by short-term alterations in metabolic parameters like blood sugar levels. Our findings provide an impetus for future experimental and clinical studies directed to investigate the mechanism of real-time blood glucose concentration on cardiac mechanics. Studies investigating other influential factors on myocardial performance in patients with diabetes should exclude the influence of short-term alterations in serum blood sugar as a potential confounder. Blood glucose levels should be measured close to the timing of the echocardiographic examination when assessing myocardial mechanics with sensible tools such as speckle tracking echocardiography.

## Abbreviations

ANOVA: analysis of variance
LV: left ventricle
T1DM: type 1 diabetes mellitus
